# Bisphenol S and Bisphenol A disrupt morphogenesis of MCF-12A human mammary epithelial cells

**DOI:** 10.1038/s41598-019-52505-x

**Published:** 2019-11-05

**Authors:** Ella Atlas, Valeria Dimitrova

**Affiliations:** 1 Environmental Health Science and Research Bureau, Health Canada, 50 Colombine Driveway, Ottawa, Canada; 2 Department of Biochemistry, Microbiology, and Immunology, University of Ottawa, Ottawa, Canada

**Keywords:** Breast cancer, Endocrine system and metabolic diseases

## Abstract

Breast cancer is one of the most common cancers diagnosed in women worldwide. Genetic predisposition, such as breast cancer 1 (BRCA1) mutations, account for a minor percentage of the total breast cancer incidences. And thus, many life style factors have also been linked to the disease such as smoking, alcohol consumption and obesity. Emerging studies show that environmental pollutants may also play a role. Bisphenol-A (BPA) has been suspected to contribute to breast cancer development, and has been shown to affect mammary gland development amongst other effects. This prompted its replacement with other bisphenol analogs such as, bisphenol-S (BPS). In this study we used the human mammary epithelial cells, MCF-12A, grown in extracellular matrix to investigate the ability of BPA and BPS to disrupt mammary epithelial cells organization. We show that both BPA and BPS were equipotent in disrupting the organization of the acinar structures, despite BPS being less oestrogenic by other assays. Further, treatment with both compounds enabled the cells to invade the lumen of the structures. This study shows that BPS and BPA are environmental pollutants that may affect mammary development and may contribute to the development of breast cancer.

## Introduction

Breast cancer is one of the most frequently diagnosed forms of cancer in women^1^. There are many factors that have been associated with breast cancer such as mutations in the breast cancer associated -1 gene, obesity, age, smoking and alcohol consumption^2^. However, an increasing body of evidence suggests that the aetiology for breast cancer may be in part related to chemical exposure. Evidence from epidemiological studies suggests that exposure to chemicals with endocrine disruptor activity, or endocrine disruptors (EDCs), may be positively correlated to the development of breast cancer especially if the exposure occurred during critical developmental stages^3^. Numerous studies have examined the correlation between exposure to environmental pollutants and development of breast cancer, in a variety of *in vitro* and *in vivo* models. The list of environmental pollutants that have been suspected to have a role in the development of breast cancer is large and growing. This includes but is not limited to polychlorinated biphenyl ethers, phthalates, triclosan, octylphenol dichlorodiphenyltrichloroethane and more^4,5^. One of the chemicals most studied to date for endocrine disrupting activities is bisphenol A (BPA). BPA is a chemical that was initially developed as an oestrogen and is now produced in large quantities and added to many consumer products such as in can linings, dental fillings and plastic bottles^6^. As a consequence, human exposure to BPA is ubiquitous. It has been reported that prenatal and perinatal exposures of rats to diethylstilboestrol (DES) or BPA altered mammary gland development and induced precancerous and cancerous lesions in the mammary gland^7,8^. Further, a study showed that perinatal exposure of rats to BPA increased the incidence of cancerous lesions in rats who also received hormone replacement therapy when they reached middle age^3^. Several studies have linked BPA exposure to increased breast cancer risk in epidemiological studies^9^. As public concerns with BPA exposure increased, industry proceeded to replace BPA with analogues such as bisphenol-S (BPS)^10^ which is now found in products labelled as BPA-free. BPS is perceived as a safer alternative to BPA based on its diminished oestrogenic activity as assessed by *in vitro* transactivation of the oestrogen receptor (ER)^11^. Recently, a study compared the endocrine disrupting activity of BPA and BPS in animals exposed postnatally to the chemicals, and found similar effects for BPA and BPS on female reproductive organs^12^. Another recent study showed that perinatal exposure to low dose BPS resulted in altered mammary gland development in female mice^13^. However, the effects of BPS on the initiation and progression of breast cancer has not yet been thoroughly assessed.

Breast cancer is a disease in which the organization of the breast epithelial cells is lost and where the cells lose polarity and proliferate out of control. It is recognised that the maintenance of the mammary gland structure and its functionality depend on the signals from the extracellular matrix, stromal cells and the neighbouring epithelial cells^14^. The 3D cell models provide an *ex vivo* approach closer to physiological conditions as compared to 2D cell culture models. Cell-cell interactions such as gap junction formation together with interactions of the cells with the extracellular matrix (ECM) provide important signals to the mammary epithelial cells that resemble the signals cells would have had *in vivo*. Bissell’s group has shown that mammary epithelial cells grown in extracellular matrix, Matrigel, differentiate into mammary gland like structures^15^. This group has also shown that when the mammary epithelial cells overexpress oncogenes the organization of the structures is disrupted and resembles cancerous lesions. Other groups have shown that stromal cells grown together with mammary epithelial cells also contribute to the cancerous phenotype adding another component to the complexity^16^. These cell-cell and cell-ECM interactions better recapitulate the epithelial cells microenvironment than 2D cultures. Further, it has been shown that the ECM contributes to tumourigenicity both by epigenetic changes that occur in the cells and by changes in signalling of receptors such as the epithelial growth factor receptor (EGF), a growth factor that has a crucial role in breast cancer^17,18^. Additionally, it has been shown that mammary epithelial cells grown in 2D differ in their gene expression profile than cells grown in 3D^19^. Mammary epithelial cells grown in 3D express genes that are otherwise lost when the cells are grown in 2D^20^. One other aspect of cells grown in 3D is the polarity, i.e. the orientation of the Golgi towards the lumen of the structure, which is maintained only in a 3D environment but not when cells are grown in monolayers^21^. Cells grown in 2D lose many of the characteristics of the organ they originated from^20^. Further, the reciprocal regulation between integrin signalling and growth factor signalling is lost in mammary epithelial 2D cultures. It is recognised now that 2D cell culture models may not have the desired predictive value for effects in humans *in vivo*. This is becoming evident for both chemical toxicity screens and drug discovery^22^. As previously mentioned this stems from the fact that the microenvironment of the cells, that includes ECM, has great influence on the gene expression and the phenotype of the cells.

For the purpose of this study we chose the non-tumorigenic spontaneously immortalized MCF-12A cell line. MCF-12A cells express both ERα and ERβ but are oestrogen independent for growth, and a recent study suggests that the ERα may not be functional in this cell line^23^. The MCF-12A cells form organized acini when grown in Matrigel, by the process previously described^24^. During the morphogenesis process, the cells form clusters with some evidence of apicobasal polarization. At around day 8 there become two evident cell populations within the developing acini: well-polarized outer cells and poorly polarized inner cells. The polarized outer cells are in direct contact with the extracellular matrix while the inner cells lack contact with the matrix. The poorly polarized cells undergo apoptosis, thus forming the hollowed out lumen on the fully developed acinus^24,25^. It has been previously established that a change in the organization of the cells in this 3D environment indicates possible carcinogenic effects^15^. In this study we examined and compared the effects of BPA and the BPA analogue BPS on the morphogenesis of MCF-12A in Matrigel in 3D.

## Results

### BPS and BPA disrupt acini organization of MCF-12A cells grown as spheroids in Matrigel

**Figure 1:**
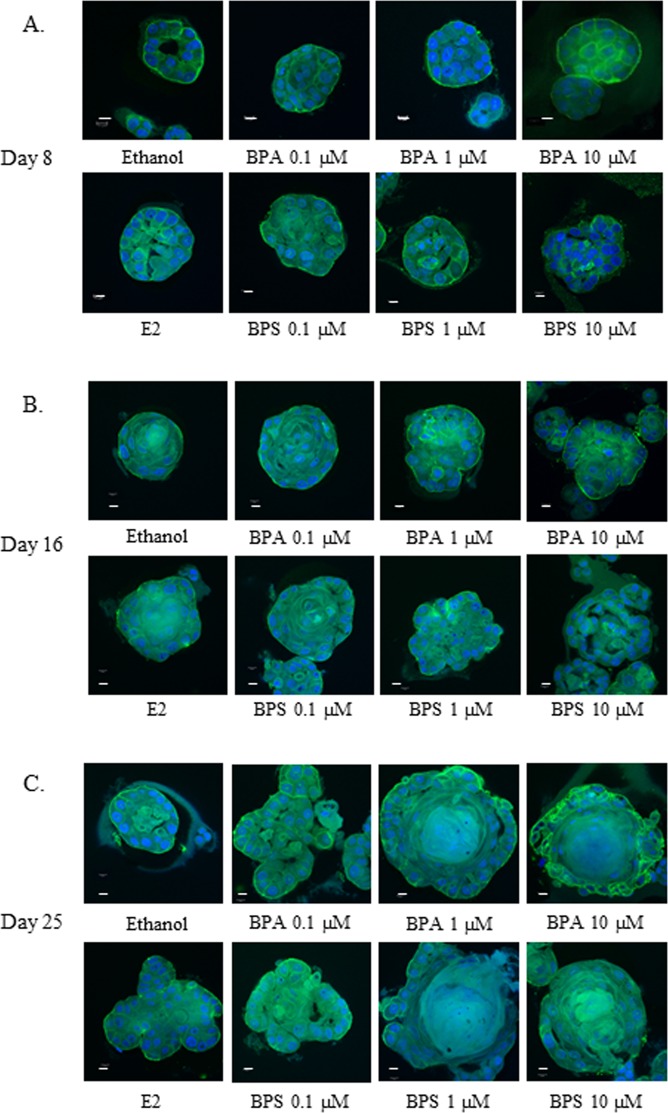
BPA and BPS disrupt acini organization of MCF-12A. MCF-12A cells were grown as acini in Matrigel. 5,000 cells per well were seeded in 8-well chamber slides as described in Material and Methods. Cells were treated every 4 days with EtOH (negative control), 1 nM E2 (positive control), or the indicated concentration of BPA and BPS and allowed to grow for 8 (**A**), 16 (**B**) and 25 days (**C**). Spheroids were immunostained with antibody against integrin-6 (green) to stain the basement membrane and counterstained with DAPI to visualize cell nuclei (blue). Confocal images are representative of three independent experiments, bars represent 10 µm.

MCF-12A cells grown in a basement membrane matrix, Matrigel, assume a 3D structure that is characterized by proliferation, membrane deposition and polarization of the cells. This process is followed by emptying of the lumen of the acinar structure by apoptosis and results in an organized mammary-like structure. We used this model system in order to evaluate whether BPA and BPS can disrupt this process in the ERα ERβ positive cell line MCF-12A. For this purpose the cells were seeded in Matrigel as described in the Material and Methods and treated with (0.1 to 10 µM) BPA and BPS. The acini were visualized using confocal microscopy at day 8 day 16 and day 25. As illustrated in Fig. 1A–C, upper panels, treatment of the cells with BPA resulted in changes in the morphology of the acini structures as early as day 8 and at concentrations as low as 0.1 µM. The cells formed globular structures and filled the lumen of the spheroids. As expected, the ethanol treated controls formed organised acini and emptied their lumen as early as day 8 (Fig. 1A). The most pronounced effects were observed after treatment of the cells for 25 days when large and disorganized acini were evident in the BPA treated acini (Fig. 1B). BPS treatment also resulted in a disruption in acini formation starting at day 8 and at all concentrations tested (Fig. 1B). BPS disrupted the organization of the cells at similar concentrations to BPA and at concentrations as low as 0.1 µM, where according to previous published data it is not capable of activating the ERs. Similar to the BPA treatments BPS effects were also detected as early as day 8 and were most pronounced at day 25. As expected, the positive control, 1 nM E2, treatment was effective at disrupting the organization of the acinar structures after treatments of the cells for 8, 16, and 25 days (Fig. 1).

### BPS and BPA increase the volume of MCF-12A cells in Matrigel

**Figure 2:**
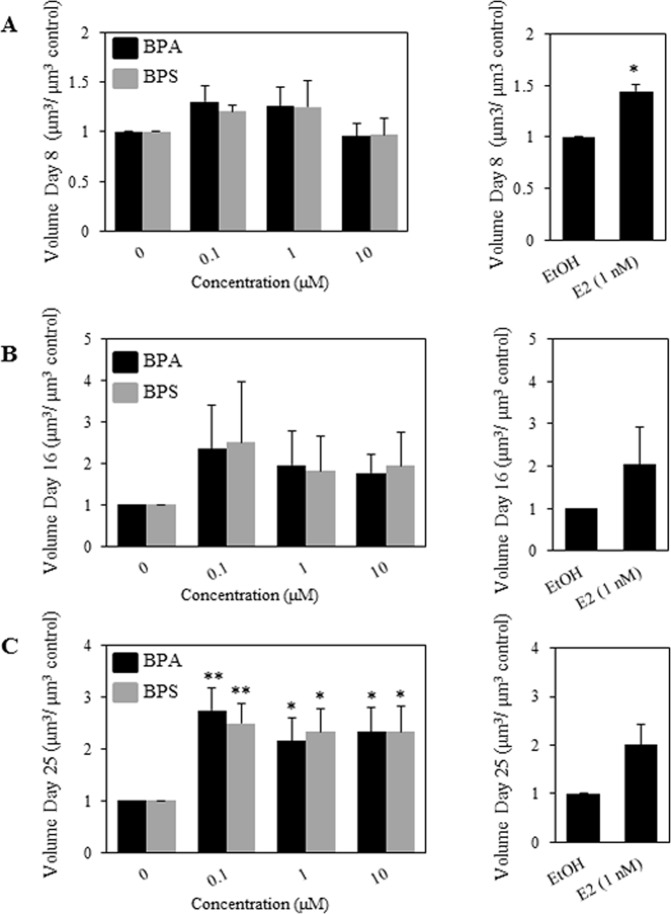
BPA and BPS increase MCF-12A acini volume. MCF-12A cells were grown as acini in Matrigel. 5,000 cells per well were seeded in 8-well chamber slides as described in Material and Methods. Cells were treated every 4 days with EtOH (negative control), 1 nM E2 (positive control), or the indicated concentration of BPA and BPS and allowed to grow for 8, 16 and 25 days. The volume of the spheroids was measured in μm^3^ at day 8 (**A**), day 16 (**B**) and day 25 (**C**). Results from 3 separate experiments, assessing at least twenty acini per condition, are graphically represented as mean volume treatment/mean volume control ± S.E.M. Statistical significance for BPA and BPS treatments was assessed by two-way ANOVA with Tukey’s post-hoc tests *denotes p < 0.05 and **denotes p < 0.01 compared to solvent control. A paired t-test was performed for the E2 condition. Acini smaller than 25 μm in diameter were excluded from the analysis, as they represent cells that did not form structures.

We proceeded to quantify the volume of the acinar structures in the ethanol treated controls, as compared to the BPA and BPS treated cells. We measured at least ten acinar structures for each condition and the results are summarized in Fig. 2. As previously indicated E2 was used as a positive control. Treatment of the MCF-12A cells grown in a 3D environment with BPA and BPS resulted in an increase in the volume of the acini trending at day 8 and 16 (Fig. 2B) and reaching statistical significance at day 25 (Fig. 2C). BPA treatment increased the volume of the acini at day 25 by 2.7-, 2.1- and 2.3- fold at 0.1, 1, and 10 µM respectively as compared to solvent treated controls. Similarly, BPS treatment resulted in increased volume of the acini at day 25 by 2.5-, 2.3- and 2.3- folds over ethanol controls at 0.1, 1, and 10 µM respectively (Fig. 2B, left panel). E2 treatment, our positive control, resulted in a 2- fold increase in the volume as compared to ethanol control (Fig. 2B,C right panels); however this increase did not reach statistical significance (Fig. 2B, right panel).

### BPS and BPA treatments results in disorganised acini with no lumen

**Figure 3:**
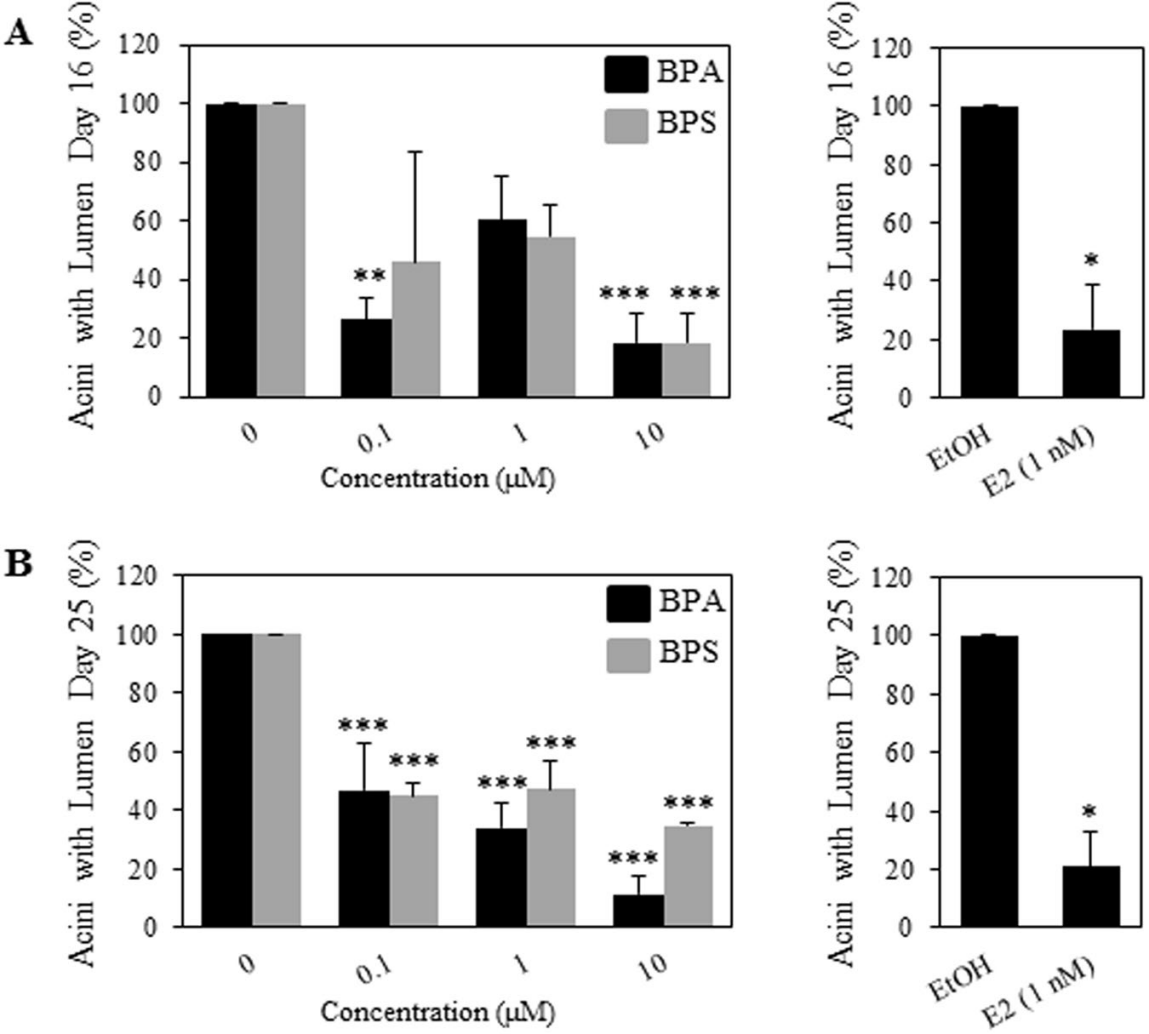
BPA and BPS treatments inhibit lumen formation of MCF-12A cells in Matrigel. Percentage (%) of acini with a lumen at day 16 (**A**) and at day 25 (**B**) was determined by analyzing confocal images. Cells were treated as described in Material and Methods with indicated concentration of BPA, BPS and E2. Structures with more than six cells present in the centre of the lumen were scored as structures with no lumen. Data corresponds to mean ± S.E.M and the results from three independent experiments, where a minimum of 20 acini were analyzed per experiment. (*p < 0.05), (**p < 0.01) and (***p < 0.001) indicate significant differences between treatments and negative control. Acini smaller than 25 μm in diameter were excluded from the analysis, as they represent cells that did not form structures.

We further characterized the acini formed by the MCF-12A cells grown in Matrigel and scored the acini for the presence of a defined lumen, void of cells in the middle of the acinar structures. We checked for lumen formation at day 16 when some of the structures should have emptied the lumen and at day 25 where most of the acini should have a formed acinus with a clear lumen (Debnath *et al*. 2003). We found that at day 16, BPA treatment reduced the number of acini with lumen by 74% and 82% at 0.1 μM and 10 μM respectively -compared to the ethanol control. BPS treatment significantly reduced the number of acini with lumen by 82% of the solvent control only at 10 μM **(**Fig. 3A). BPA decreased the percent acini with lumen at day 25, at all concentrations, 0.1, 1 and 10 μM, by 63%, 76% and 89% respectively (Fig. 3B). BPS was also able to decrease percent acini with lumen as compared to ethanol controls by 65%, 63% and 76% at 0.1, 1 and 10 μM respectively (Fig. 3B).

### BPS, BPA or E2 have no effect on proliferation rates of the MCF-12A cells grown as monolayers

**Figure 4:**
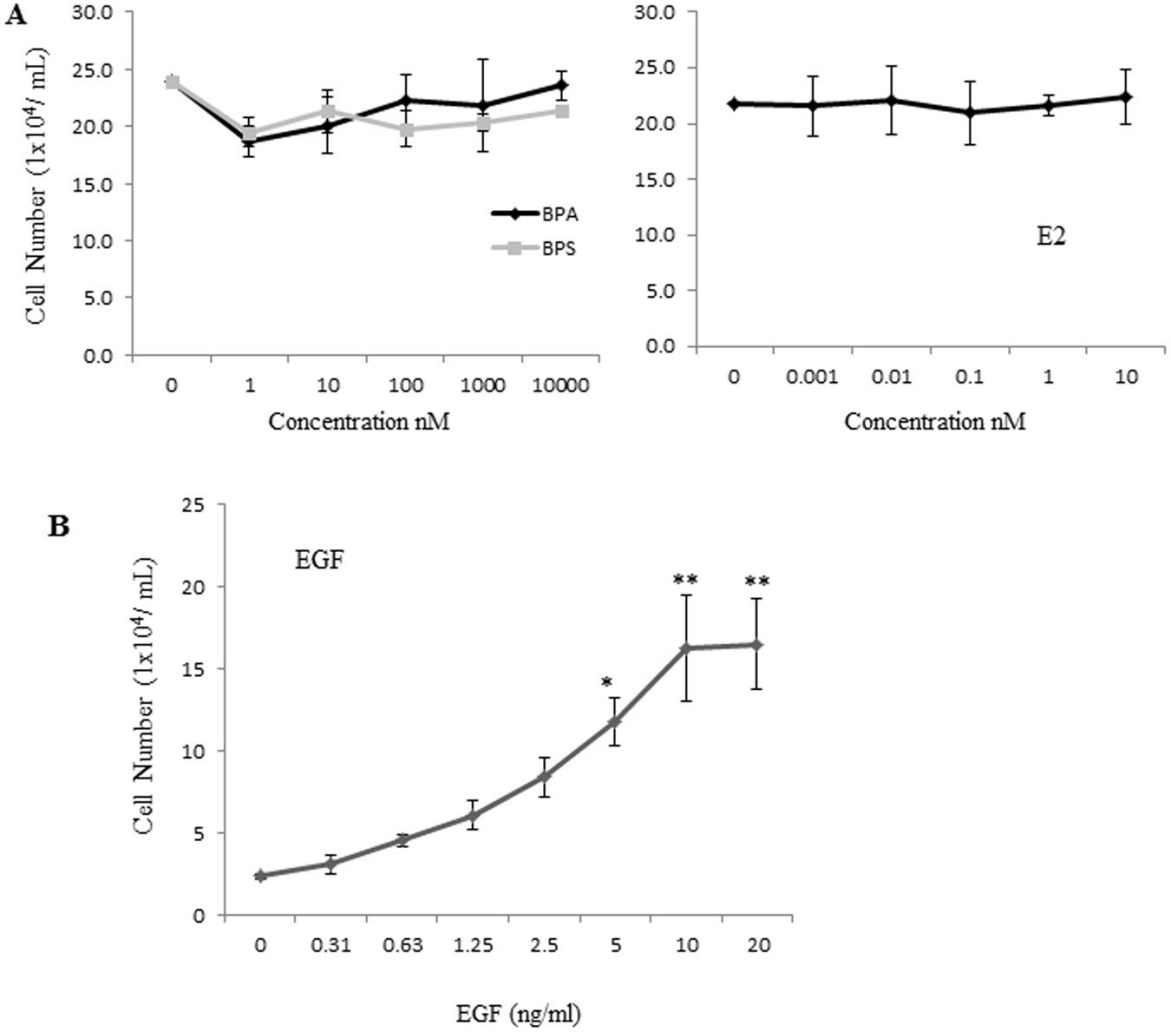
Proliferation of MCF-12A cells treated with BPA, BPS and E2. MCF-12A cells were seeded at 20,000 cells per well in a 24-well plate in MCF-12A 5% charcoal stripped horse serum, 0.5 μg/mL hydrocortisone, 100 ng/mL cholera toxin, 10 μg/mL insulin, 1X pen/strep and phenol red free DMEM/F12 media and treated with the indicated chemicals every two days. Cells were counted after seven days of being treated with indicated concentration of BPA, BPS and E2 (**A**) or the indicated concentrations of EGF (**B**). Data corresponds to mean ± S.E.M from three independent experiments done in triplicates.

Based on the results in 3D cultures described above we further investigated whether BPS and BPA enhance the proliferation of the MCF-12A cells when the cells are grown as monolayers. For this purpose, cells were seeded as monolayers and treated with BPS, BPA E2 and ethanol at the indicated concentrations for 48 hours followed by cell counting. The results presented in Fig. 4 show that there was no change in the proliferation rate of the MCF-12A cells in response to BPS, BPA or E2 (Fig. 4A). We also confirmed that MCF-12A cells are dependent on epidermal growth factor (EGF) for their growth as monolayers (Fig. 4B). Together these results show that BPA and BPS do not affect cell proliferation of these cells when grown as monolayers.

### BPA and BPS have different potencies when tested for the growth of the oestrogen dependent cell line MCF-7

**Figure 5:**
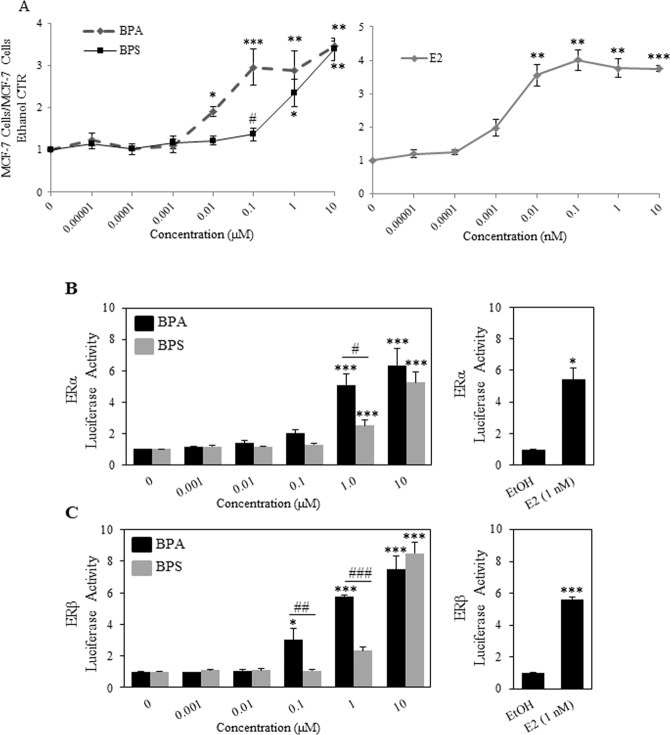
BPA and BPS have estrogenic activity. (**A**) The estrogen dependent MCF-7 cells were seeded at 20,000 cells per well in a 24-well plate and incubated at 37 °C and 5% CO2 in 5% charcoal stripped FBS and phenol red free DMEM/F12 media. Cells were counted at day seven after being treated every 2 days with indicated concentration of BPA, BPS and E2 (**A**). Data correspond to mean ± S.E.M from three independent experiments done in triplicates. Statistical significance for BPA and BPS treatments was assessed by two-way ANOVA with Tukey’s post-hoc tests *denotes p < 0.05 **denotes p < 0.01 and ***denotes p < 0.001 compared to solvent control. ^#^Denotes p < 0.05 BPS versus BPA. (**B**,**C**) COS-7 cells were transfected as described in the Materials and Methods with VP16-ERα (**B**) or VP16-ERβ (**C**) 3X ERE-TATA-Luc, and pCMV-RL and treated with increasing amounts of BPS or BPA (0.001–10 µM). 24 hours after treatment reporter gene activity was determined. Data represent the mean ± S.E.M of three independent experiments. Significantly different (*p < 0.05, **p < 0.01, ***p < 0.001) reporter gene activity was determined relative to transfected vehicle-treated cells using a one-way ANOVA followed by Tukeys post-hoc analysis.

It has been previously shown by others that BPA is more oestrogenic than BPS. Our results from Figs 1–3 show that BPS is equal to BPA in increasing acini volumes and diminishing lumen formation of the MCF-12A cells grown in Matrigel as both chemicals show effects at concentrations as low as 100 nM, whereas BPS has been shown not to be oestrogenic at such low concentration. For this purpose, we used the E2 dependent cell line MCF-7 that requires E2 for its growth^26^. The results in Fig. 5 show that the MCF-7 cells started to proliferate in response to 1 pM E2 and reached a plateau at 10 nM E2. As expected, BPA promoted MCF-7 cell proliferation starting at 100 nM and BPS was active in this model system only at 1 μM (Fig. 5). These results are in concordance with other studies showing that BPS is less oestrogenic than BPA by a factor of 10.

### BPA and BPS enhance the transcriptional activity of ERα and ERβ

**Figure 6:**
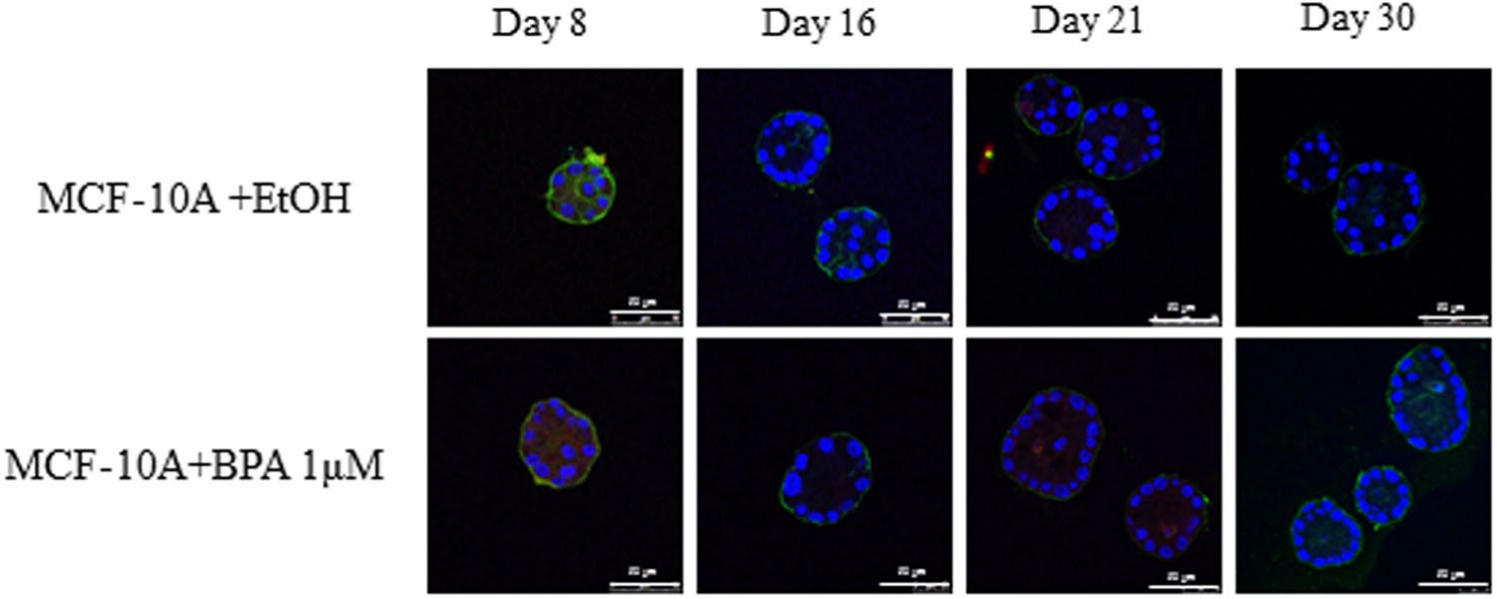
BPA BPS or E2 did not affect MCF-10A cells growth in 3D. (**A**) MCF-10A cells, expressing ERβ, were treated with 1 µM BPA or ethanol control for 8, 16, 21 and 30 days in Matrigel as described in the Material and Methods, fixed and stained with an antibody against pan-actin (green) or DAPI (blue) to stain nuclei.

MCF-12A cells express both ERα and ERβ. We asked whether the two chemicals, BPA and BPS, are able to activate the ERα and ERβ in a luciferase assays using expression vectors for ERα or ERβ and a construct containing the luciferase gene under the control of oestrogen responsive elements (EREs). We found that, similarly to the results obtained with MCF-7 cells, BPA was able to transactivate the ERα starting at 100 nM (2 fold) and reaching a maximum of 6 fold at 10 μM when compared to ethanol control (Fig. 6A). BPS was able to transactivate the receptor only at 1 µM (2.5 fold) and at 10 µM transactivated the receptor by 5 fold as compared to the ethanol control. The ERβ transactivation by the chemicals followed a similar trend. At 100 nM BPA was able to transactivate the receptor by 3 fold as compared to solvent control (Fig. 6B) and by 6 and 7.5 fold compared to ethanol control at 1 and 10 µM respectively. BPS was active only starting at 1 µM similarly to its activity on the ERα.

### MCF-10A cells growth in 3D is not affected by BPA and BPS

**W**e treated MCF-10A cells, another non-tumorigenic cell line, that express ERβ but do not express ERα, with 1 µM BPA for 8, 16, 25 and 30 days or with solvent control. Figure 6 shows that BPA had no effect on the size or organization of the acini formed by the MCF-10A cells even after repeat treatment for 30 days, suggesting that the ERα may be required for the effects.

## Discussion

The mammary gland is one of the targets for endocrine disruption. Studies in mice have shown that gestational exposure of female mice to BPA resulted in alterations in the reproductive organs of the animals altered mammary gland development and subsequent susceptibility to mammary tumour formation later in life^3,8^. Since BPA is being replaced with other analogues, such as BPS, it is important to determine whether these analogues have the potential to disrupt mammary gland development and/or have a potential to increase breast cancer incidence. However, performing lactational and prenatal exposure studies for all the analogues is not feasible. The MCF-12A and 10 A grown in 3D structures, in extracellular matrix, have proven to be a useful model to examine key molecular events in breast carcinogenicity *in vitro*^27^. We have used this model in order to examine the potential of BPA and BPS to affect human mammary epithelial cells and their ability to form mammary gland-like structures when embedded in extracellular matrix. The MCF-12A cell line has been shown to express both ERs but the responsiveness of the cells to the hormone has remained ambiguous. In fact, a recent study showed that the receptor may not be functional in these cells due to their inability to proliferate in response to oestrogen^23^. Given that BPA is able to transactivate the ERα at 100 nM and that BPS was not found to be oestrogenic at this concentration we used 100 nM as the starting concentration. Both BPA and BPS disrupted the morphogenesis of the MCF-12A cells grown in 3D starting at 100 nM. This was surprising because we and others have shown that at a concentration of 100 nM BPS was unable to support the growth of the E2 dependent cell line MCF-7 or transactivate the ERs in luciferase assays^28^. This suggests that the 3D environment may deliver signals through the integrins or other molecules that are in contact with the extracellular matrix that render the cells more susceptible to the effects of these chemicals. This has been previously shown to be the case for sensitivity to chemotherapeutic drugs where cells grown in 3D showed enhanced sensitivity to chemotherapy^29^. Another possibility is that BPS and BPA may activate other membrane bound receptors such as G protein-coupled oestrogen receptor 1 (GPER) and that may be the receptor that is targeted in the 3D environment. In fact GPER was shown to be a target for BPA; however the concentrations used in that study were much higher than the ones used in this study^30,31^. It is not known whether BPS can also activate GPER, however there is one publication showing that the receptor may be involved in BPS mediated effects^32^. Interestingly, ICI co-treatment of the cells resulted in more spherical structures but only partially decreased the size of the acini (data not shown), suggesting that ERα may still be involved and that it may be only one of the receptors that are mediating the effects of BPA and BPS. We and others have shown that BPS, in contrast to BPA, is unable to transactivate the ERs at 0.1 µM and significantly activated the oestrogen receptors starting only at 10 µM, by the assays used in this study. Surprisingly, 0.1 µM is a concentration where we observed effects on the size of the acini and the lumen formation as early as day 16 of treatment with both chemicals. This may indicate that BPS may have other molecular targets, as previously shown by RNAseq experiments in other systems^33^. One other possibility is that the ERβ may be involved. Both chemicals can activate the ERβ receptor, albeit at similar concentrations as for the ERα, i.e. 0.1 µM for BPA and 10 µM for BPS. However, this receptor is known to be antiproliferative and thus it is extremely unlikely that its activation will disrupt the acinar morphogenesis^34^.

Both BPA and BPS disrupted mammary epithelium organization into mammary gland like structures and enabled the cells to populate the lumen. This may indicate that the chemicals are able to affect the tumourigenicity of the cells. The MCF-12A cells are luminal epithelia cells that were isolated from a benign growth. They are unable to grow as tumours in nude mice and are unable to form colonies in agar, markers of a tumorigenic phenotype, and form organised acini structures in extracellular matrix^35,36^. The fact that treatment of the cells with BPA and BPS at concentrations as low as 0.1 µM enabled the cells to fill the lumen of the acini and disrupt the acinar organization may indicate that these chemicals are affecting breast cancer progression or initiation since the ability of the cells to fill the lumen is one of the hallmarks of breast carcinoma *in situ*^37^. It is known that some luminar epithelial cells can progress through the epithelial-mesenchymal transition to become invasive metastatic tumours^21^. It is not clear from this study if these chemicals are able to promote this transition, however this warrants further investigation. The effects in response to 100 nM BPS on acinar organization and growth in the 3D environment were not detectible when the cells were adherent to plastic (normal growth conditions). Our results show that both BPA and BPS disrupted the organization of the acinar structures starting at the same concentrations despite the fact that BPS was able to activate the receptors at far higher concentrations than BPA (0.1 µM for BPA and 10 µM for BPS). Further, EGF promoted the growth of the MCF-12A cells under standard growth conditions when MCF-12A cells were grown as monolayer cultures, but oestrogen or BPA and BPS had no effect. This study shows that the extracellular matrix interactions may change the susceptibility of the cells to environmental pollutants and the effects on endpoints such as mammary gland organization and invasion cannot be determined in 2D conditions. Thus, the development and understanding of such *in vitro* assays for evaluating the effects of the environment on the mammary gland are of great importance moving forward.

## Materials and Methods

### Reagents

All the materials were purchased from Sigma Aldrich Inc. (Oakville, ON) unless otherwise specified.

### Cell culture

MCF-12A, MCF-10A and MCF-7 were purchased from the ATCC. MCF-12A and MCF-10A cell lines were maintained in DMEM/F12 (Thermo Fisher Scientific) supplemented with 5% horse serum (Invitrogen), 20 ng/mL EGF (Invitrogen), 0.5 μg/mL hydrocortisone, 100 ng/mL cholera toxin, 10 μg/mL insulin (Roche) and 1X pen/strep (Wisent). The MCF-7 cell line was maintained in DMEM/F12 with 10% fetal bovine serum (Wisent) and 1X pen/strep.

### 3D Matrigel culture

MCF-10A and MCF-12A cells were cultured in Matrigel following an adapted protocol from Debnath *et al*.^24^. Briefly, 8-well chamber permanox slide (Thermo Scientific) was coated with Growth Factor Reduced Matrigel Matrix, (Matrigel), (BD Biosciences). 5000 MCF-12A cells were seeded in DMEM/F12 phenol red free media supplemented with 2% charcoal stripped horse serum (Invitrogen), 5 ng/mL EGF (Invitrogen), 0.5 μg/mL hydrocortisone, 100 ng/mL cholera toxin, 10 μg/mL insulin (Roche), 1X pen/strep (Wisent) and 2% Matrigel. MCF-12A cells were treated with BPA or BPS (0.1, 1, and 10 μM) or 1 nM oestrogen (E2) (Sigma) or 0.1% ethanol. The treatment media was changed every 4 days and the cells were grown until time points at days 8, 16 and 25. For the experiments using ICI 182,780 (Sigma), the cells were plated in Matrigel as described above in the presence or absence of 1 µM ICI 182,780.

### Immunostaining and confocal microscopy

The cells were extracted from the Matrigel as previously described and fixed with 4% paraformaldehyde (EM grade, Electron Microscopy Sciences). Slides were permeabilized with 0.5% Triton and washed with PBS/Glycine. Then the slides were blocked with 10% goat serum (Wisent) and 20 µg/mL goat anti-mouse F(ab)’ fragment (Cedarlane). The acini were stained with antibody against Integrin (Millipore) and nuclear staining with DAPI (Invitrogen). Prolong Gold Antifade reagent (Life Technologies) was added to each well. Confocal fluorescence images were taken using a Leica TCS SP8 confocal microscope (Leica Microsystems).

### Cell proliferation assay

MCF-7 and MCF-12A cells were grown in monolayer proliferation assays as previously described^26^. Briefly, MCF-12A or MCF-7 cells were seeded in a 24-well plate in DMEM/F12 phenol red free media supplemented with 5% charcoal stripped fetal calf serum or 2% charcoal stripped horse serum, 5 ng/ml EGF, 5 ng/mL EGF, 0.5 μg/mL hydrocortisone, 100 ng/mL cholera toxin, 10 μg/mL insulin respectively. Cells were treated with ethanol control, E2 in increments of 10 fold from 0.01pM to 10 nM, and BPA and BPS in increments of 10 fold from 0.01 nM to 10 μM. The media was replenished every two days until day seven when the cells were counted in a TC20 Automated Cell Counter (Bio-Rad).

### Reporter gene assay

COS-7 cells were seeded in phenol red-free DMEM (Wisent) supplemented with 5% dextran-coated charcoal stripped serum (Sigma-Aldrich). Twenty-four hours after plating, cells were transfected with plasmid DNA using Fugene HD (Promega) according to the manufacturer’s recommendations. For the transcriptional assays, cells were transfected with 10 ng of pRL-CMV (renilla; internal control, Promega) 25 ng of VP-16-ERα 25 ng of VP-16-ERβ (Addgene^38^ and 125 ng of 3X ERE-TATA-luciferase (ERE-luc) (Addgene^38^. Twenty-four hours after transfection, cells were treated with vehicle control and the indicated concentrations of BPS or BPA, as well as 1 nM E2 as positive control. Twenty-four after treatment, cells were lysed using 1X Passive Lysis Buffer (Promega). Luciferase activity was quantified with the Dual Luciferase Assay kit (Promega) using the Glomax96 Luminometer (Promega). Luciferase activity was normalized to Renilla levels and to vehicle control.

### Statistical analysis

A two-way Analysis of Variance (ANOVA) followed by Tukey’s multiple comparison tests was performed where applicable. For estrogen treatment compared to control student *t*-test was used.

The datasets generated during and/or analysed during the current study are available from the corresponding author on reasonable request
